# Impact of estrogen receptor levels on outcome in non-metastatic triple negative breast cancer patients treated with neoadjuvant/adjuvant chemotherapy

**DOI:** 10.1038/s41523-021-00308-7

**Published:** 2021-08-02

**Authors:** Maria Vittoria Dieci, Gaia Griguolo, Michele Bottosso, Vassilena Tsvetkova, Carlo Alberto Giorgi, Grazia Vernaci, Silvia Michieletto, Silvia Angelini, Alberto Marchet, Giulia Tasca, Elisa Genovesi, Enrico Cumerlato, Marcello Lo Mele, PierFranco Conte, Valentina Guarneri

**Affiliations:** 1 Division of Oncology 2, Istituto Oncologico Veneto IRCCS, Padova, Italy; 2 Department of Surgery, Oncology and Gastroenterology, University of Padova, Padova, Italy; 3 Department of Pathology, Azienda Ospedaliera di Padova, Padova, Italy; 4 Breast Surgery Unit, Istituto Oncologico Veneto - IRCCS, Padova, Italy; 5 Clinica Chirurgica 1, Azienda Ospedaliera di Padova, Padova, Italy

**Keywords:** Breast cancer, Prognostic markers, Breast cancer, Breast cancer, Tumour biomarkers

## Abstract

Although 1% is the recommended cut-off to define estrogen receptor (ER) positivity, a 10% cut-off is often used in clinical practice for therapeutic purposes. We here evaluate clinical outcomes according to ER levels in a monoinstitutional cohort of non-metastatic triple-negative breast cancer (BC) patients undergoing (neo)adjuvant chemotherapy. Clinicopathological data of 406 patients with ER < 10% HER2-negative BC treated with (neo)adjuvant chemotherapy between 01/2000 and 04/2019 were collected. Patients were categorized in ER-negative (ER < 1%; *N* = 364) and ER-low positive (1–9%, *N* = 42). At a median follow-up of 54 months, 88 patients had relapsed and 64 died. No significant difference was observed in invasive relapse-free survival (iRFS) and overall survival (OS) according to ER expression levels, both at univariate and multivariate analysis (5-years iRFS 74.0% versus 73.1% for ER-negative and ER-low positive BC, respectively, *p* = 0.6; 5-years OS 82.3% versus 76.7% for ER-negative and ER-low positive BC, respectively, *p* = 0.8). Among the 165 patients that received neoadjuvant chemotherapy, pathological complete response rate was similar in the two cohorts (38% in ER-negative, 44% in ER-low positive, *p* = 0.498). In conclusion, primary BC with ER1–9% shows similar clinical behavior to ER 1% BC. Our results suggest the use of a 10% cut-off, rather than <1%, to define triple-negative BC.

## Introduction

Estrogen receptor (ER) expression, as determined by immunohistochemistry (IHC), is a key prognostic and predictive biomarker in breast cancer (BC) and its evaluation is mandatory in all cases of invasive disease^1^. However, the definition of a clinically relevant cut-off for ER positivity remains a controversial topic.

In 2010, a joint guideline of the American Society of Clinical Oncology and the College of American Pathologist (ASCO/CAP) defined ER-positive tumors as tumors expressing ER by IHC in ≥1% of BC cells^2^. This threshold was adopted based on studies reporting some degree of benefit from adjuvant endocrine therapy in patients with ER staining as low as 1%^3^.

Although most BCs show either complete absence of ER staining (0%) or ER positivity in ≥10% of tumor cells, a small subgroup of BCs presents low levels of ER expression (i.e., defined by 1–9% of ER^+^ stained cells). These ER low-expressing tumors have been reported to present biological features similar to ER-negative tumors and are mostly classified as basal-like or HER2-enriched by PAM50 intrinsic subtyping^4–6^. Moreover, there have been some reports that BCs with low-ER levels might present a clinical behavior more similar to ER-negative than to ER-positive BCs, both in terms of response to neoadjuvant chemotherapy and prognosis^6–8^. These data suggest that the use of a threshold of 1% stained BC cells to define ER-positive status might not accurately reflect the underlying biological nature and clinical behavior of the tumor.

Acknowledging this limitation, the St. Gallen Consensus 2015 reported that ER expression values between 1 and 9% should be considered equivocal and that endocrine treatment alone, in the absence of chemotherapy, should not be considered a reliable adjuvant treatment for these patients. Consistently, the 2020 ASCO/CAP update introduced a new reporting category for BCs with 1–10% cells staining positive for ER, which should be reported as ER-low positive with a recommended comment^9^. Although relatively uncommon (representing 2–3% of ER-positive BCs), these invasive BCs with low ER-positivity represent a clinical challenge as only limited data regarding their optimal treatment is available. Moreover, the use of a restrictive threshold for ER-positivity could prevent the inclusions of patients with low ER-positivity in clinical trials testing the use of new targeted agents (e.g., immunotherapy), therefore leading to the lack of clinical evidence for the use of novel agents in this subgroup of patients. For this reason, some pragmatic trials testing the use of immunotherapy in triple-negative BC (e.g., A-BRAVE trial, TONIC trial)^10,11^ have in fact applied a 10% threshold to define ER-positivity.

In this study, we evaluate clinical outcomes of ER-low positive BCs as compared to ER-negative BCs in a monoinstitutional cohort of patients with non-metastatic ER < 10% breast cancer undergoing (neo)adjuvant chemotherapy.

## Results

### Patient characteristics and treatments received

A total of 406 patients with stages I–III BC with ER expression <10% by IHC (and HER2-negative) treated with neoadjuvant and/or adjuvant chemotherapy were included in the analysis (Supplementary Fig. 1): 364 (90%) with ER-negative (ER < 1%) BC and 42 (10%) with low ER expression (ER1–9%) BC.

Among these 406 patients, 134 (33%) had stage I disease, 212 (52%) had stage II disease and 59 (15%) had stage III disease. Most patients (365, 90%) had invasive carcinoma of no special type and grade 3 tumors (351, 88%). As by inclusion criteria, all patients included in the analysis received chemotherapy, either in the neoadjuvant or in the adjuvant setting: almost half (41%, *N* = 165) received neoadjuvant treatment, while 274 patients (67%) received adjuvant chemotherapy (41 after previous neoadjuvant treatment). Most patients (298; 73%) received both an anthracycline and a taxane agent as part of the neoadjuvant and/or adjuvant treatment. Only 19 patients (5%) received any kind of adjuvant endocrine treatment.

The IHC protocol used for ER analysis by the reference pathology department for the Istituto Oncologico Veneto for BC patients was changed in January 2012; however, no significant difference in the prevalence of low ER expression (ER1–9%) BC before 2012 (12.4%, 18/145) and after 2012 (9.2%; 24/261) was observed (*p* = 0.308).

### Patient characteristics and treatments according to ER expression

Patient demographic and clinicopathological characteristics by ER expression subgroup are reported in Table 1.

**Table 1:** Baseline patient characteristics and treatment received.

	Total (*N* = 406)	ER < 1% (*N* = 364)	ER1–9% (*N* = 42)	*p*-value
Median age, years (range)	54 (25–84)	55 (25–84)	51 (30–82)	0.136
Histology				
No special type	365 (90%)	324 (90%)	41 (98%)	0.154^a^
Lobular	17 (4%)	16 (4%)	1 (2%)	
Apocrine	8 (2%)	8 (2%)	0	
Metaplastic	6 (1%)	6 (2%)	0	
Medullary	5 (1%)	5 (1%)	0	
Clinical stage at diagnosis				
I	134 (33%)	126 (39%)	8 (19%)	0.031
II	212 (52%)	188 (52%)	24 (57%)	
III	59 (15%)	49 (13%)	10 (24%)	
Histologic grade				
G1–G2	47 (12%)	43 (12%)	4 (10%)	0.803
G3	351 (88%)	314 (88%)	37 (90%)	
Median Ki67 expression % (range)	58 (1–90)	56 (1–90)	60 (5–90)	0.170
Neoadjuvant chemotherapy				
Yes	165 (41%)	141 (39%)	24 (57%)	0.033
No	241 (59%)	223 (61%)	18 (43%)	
Type of neoadjuvant chemotherapy				
Anthracycline-taxane based	101 (61%)	85 (60%)	16 (67%)	0.714
Anthracycline-taxane-based plus platinum salts	64 (39%)	56 (40%)	8 (33%)	
Adjuvant chemotherapy				
Yes	274 (67%)	251 (69%)	23 (55%)	0.092
No	132 (33%)	113 (31%)	19 (45%)	
Chemotherapy received (in neoadjuvant and/or adjuvant setting)				
Anthracycline-taxane based	211 (52%)	185 (51%)	26 (62%)	-
Anthracycline-taxane-based plus platinum salts	87 (21%)	77 (21%)	10 (24%)	
Taxane based	25 (6%)	23 (6%)	2 (5%)	
Taxane-platinum based	8 (2%)	8 (2%)	0 (0%)	
Other^b^	75 (18%)	71 (20%)	4 (10%)	
Adjuvant endocrine treatment				
Yes	19 (5%)	13 (4%)	6 (14%)	0.002
No	387 (95%)	351 (96%)	36 (86%)	

^a^Fisher exact test of invasive carcinoma of no special type versus other histology in the ER < 1% and ER1–9% cohorts.

^b^Mostly CMF.

No significant difference regarding age at BC diagnosis, histologic grade, and proliferative index (ki67) was observed between ER-low positive (ER 1–9%) and ER-negative tumors. Special type histology was more frequently observed among ER-negative tumors (10%) than among tumors with low ER expression (2%), although the difference was not statistically significant (*p* = 0.154). Among ER-low positive tumors, 57% were progesterone receptor (PR)-negative and 43% PR-low positive (defined as PR expression 1–9% by IHC), while no case of PR-positive BC was observed. Among ER-negative, 97% were PR-negative, 2.7% PR-low positive and only one case presented a PR expression of 10%.

In our cohort of patients, patients with ER-low positive BCs presented at a significantly more advanced disease stage as compared to patients with ER-negative tumors (stage I 19% versus 39% and stage III 24% vs 13%, respectively; *p* = 0.031). Consistently, more patients received neoadjuvant chemotherapy in the ER-low positive cohort as compared to the ER-negative cohort (57% versus 39%, *p* = 0.033).

No significant difference was observed in the type of chemotherapy regimens applied: patients receiving both an anthracycline and a taxane agent as part of the neoadjuvant and/or adjuvant treatment were 86% of patients with ER-low positive tumors and 72% of patients with ER-negative tumors, respectively (*p* = 0.131).

As expected, adjuvant endocrine therapy use was significantly more frequent in the ER-low positive subgroup (15% vs 4%, *p* = 0.002).

### Prognostic impact of ER expression

At a median follow-up of 54 months, 88 patients had an invasive relapse and 64 had died.

**Fig. 1:**
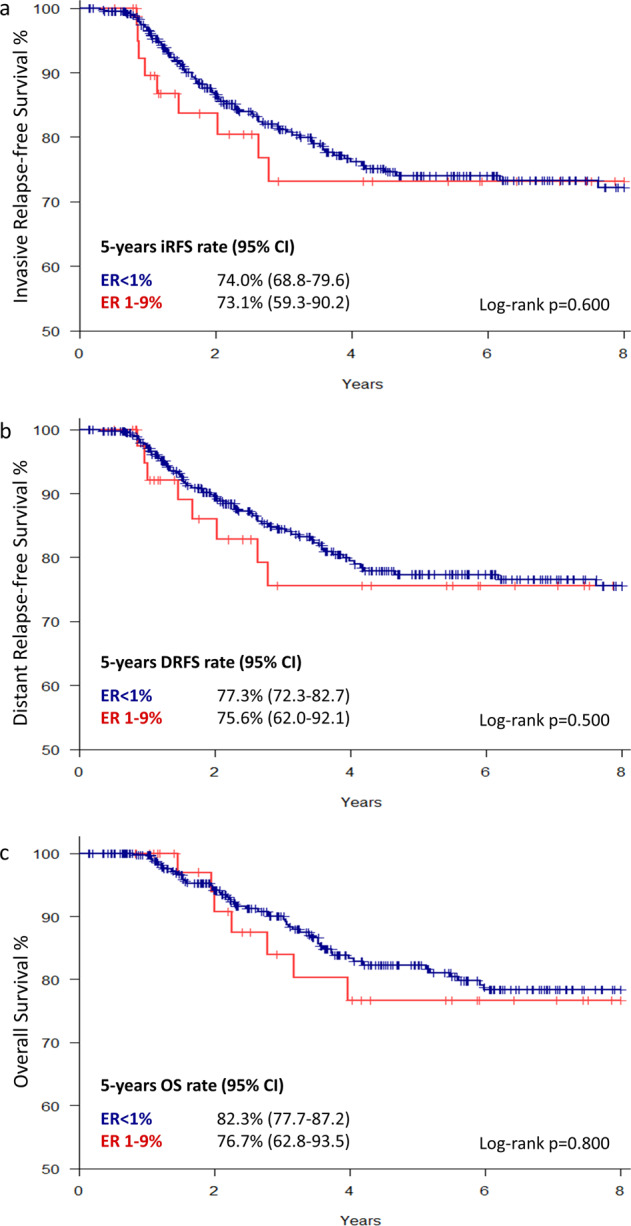
Survival outcomes according to ER levels. **a** Invasive relapse-free survival according to ER levels. **b** Distant relapse-free survival according to ER levels. **c** Overall survival according to ER levels.

5-years invasive relapse-free survival (iRFS) was 73.9% (95% confidence interval (95% CI) = 69.0–79.2%) in the whole population, 74.0% (95% CI = 68.8–79.6%) for patients with ER-negative BC and 73.1% (95% CI = 59.3–90.2%) for patients with ER-low positive BC. No significant difference according to ER expression levels was observed (log-rank *p* = 0.600; Fig. 1a).

No significant difference according to ER expression levels was observed for distant relapse-free survival (DRFS) (5-years DRFS 77.3% versus 75.6% for patients with ER-negative and ER low positive BC, respectively; log-rank *p* = 0.500; Fig. 1b) and overall survival (OS) (5-years OS 82.3% versus 76.7% for patients with ER-negative and ER low positive BC, respectively; log-rank *p* = 0.800; Fig. 1c).

At univariate Cox model analysis in the overall study cohort, disease stage at diagnosis was the only clinicopathological variable significantly associated with iRFS, while disease stage and age at diagnosis were significantly associated with DRFS and OS. ER expression levels (both when evaluated as a continuous variable and when separated in the two subgroups ER < 1% and ER 1–9%) did not significantly impact iRFS, DRFS, and OS both at univariate analysis and after correction by significant clinicopathological variables (Table 2).

**Table 2:** Cox models for iRFS, DRFS, and OS (univariate and multivariate).

	iRFS		DRFS		OS	
	Univariate	Multivariate	Univariate	Multivariate	Univariate	Multivariate
	HR (95% CI)	HR (95% CI)	HR (95% CI)	HR (95% CI)	HR (95% CI)	HR (95% CI)
Age (continuous)	1.01 (0.99–1.03)	–	1.02 (1.00–1.04)	1.03 (1.01–1.04)	1.02 (1.00–1.04)	1.03 (1.01–1.04)
Grades						
G1–G2	Ref	–	Ref	–	Ref	–
G3	1.00 (0.54–1.86)	–	1.08 (0.55–2.11)	–	1.65 (0.70–3.85)	–
Ki67 baseline (continuous)	1.00 (0.99–1.01)	–	0.99 (0.98–1.00)	–	1.00 (0.99–1.01)	–
Stages						
I	Ref	Ref	Ref	Ref	Ref	Ref
II	1.93 (1.07–3.47)	1.93 (1.07–3.48)	1.45 (0.79–2.67)	1.67 (0.90–3.09)	1.33 (0.70–2.51)	1.53 (0.81–2.93)
III	4.59 (2.45–8.60)	4.60 (2.44–8.65)	4.01 (2.12–7.57)	4.73 (2.48–9.02)	3.02 (1.53–5.99)	3.59 (1.79–7.21)
ER expression (continuous)	1.00 (0.78–1.28)	–	1.04 (0.80–1.34)	–	1.02 (0.77–1.36)	–
ER expression						
<1%	Ref	Ref	Ref	Ref	Ref	Ref
1–9%	1.20 (0.62–2.32)	0.98 (0.51–1.91)	1.26 (0.63–2.52)	1.21 (0.59–2.47)	1.10 (0.50–2.40)	1.06 (0.48–2.36)

### ER expression and pCR in patients treated with neoadjuvant chemotherapy

Pathologic complete response (pCR) was achieved in 63 out of 165 patients (38%) receiving neoadjuvant chemotherapy: 10 patients with ER low positive BC (10/24, 44%) and 53 patients with ER-negative BC (53/141, 38%). pCR rates were similar in the two subgroups (*p* = 0.498).

**Fig. 2:**
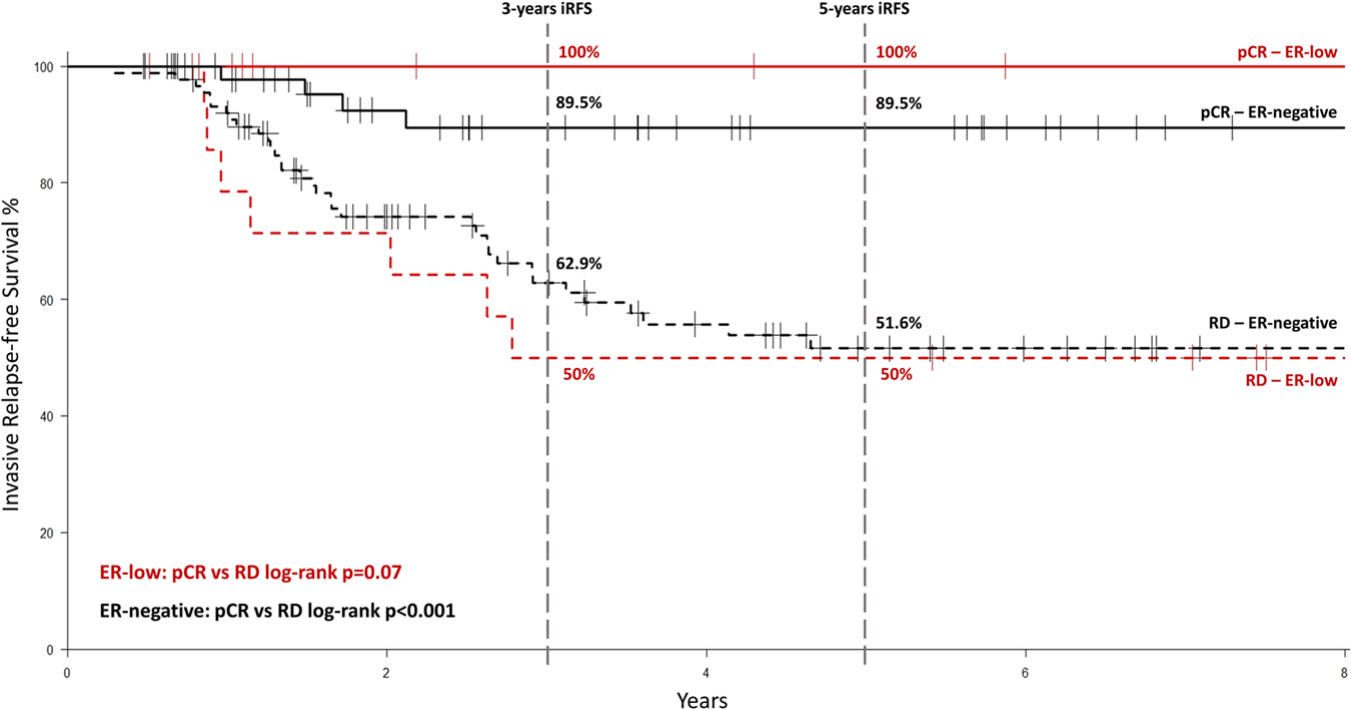
Invasive relapse-free survival according to ER-status and response to neoadjuvant chemotherapy. Invasive relapse-free survival according to ER-status (ER-low positive BC in red and ER-negative BC in black) and response to neoadjuvant chemotherapy (pCR pathological complete response in solid line; RD residual disease in dashed line).

In both subgroups, pCR was associated with long-term outcome (5-year iRFS 100% for pCR versus 50% for residual disease and 89.5% for pCR versus 51.6% for residual disease in the ER 1–9% and ER < 1% subgroups, respectively) and relapses occurred early, mostly in the first 3 years after BC diagnosis (Fig. 2). No patient with ER low positive BC achieving pCR received adjuvant endocrine treatment.

## Discussion

BC is a clinically heterogeneous disease and ER status determined by IHC is an important prognostic factor as well as a predictor of response to endocrine treatment. ER-low positive status, defined as 1–9% tumor cells positively stained for ER, is a challenging scenario with limited evidence regarding the clinical characteristics and prognosis of this subgroup of BC patients.

This monoinstitutional study describes clinicopathological characteristics, treatments, and outcomes of ER-low positive stage I–III BC and compares them with those of their ER-negative counterparts.

In this cohort of patients, ER-low positive BCs presented clinical-pathological characteristics at least as aggressive as those reported for ER-negative BCs. In fact, both presented similarly high Ki67 levels, high histological grade, and relatively young age at cancer diagnosis, with no significant differences between subgroups. Moreover, ER-low positive BCs did not present a more favorable stage at diagnosis as compared with ER-negative BCs. On the contrary, both subgroups presented with locally advanced stage at diagnosis in a consistent percentage of cases and, in this cohort, ER-low positive BCs presented an even higher frequency of locally advanced stages at diagnosis as compared to ER-negative tumors, although this difference has no clear biological explanation and might be a casual observation due to the small size of the ER-low positive subgroup.

Overall, these results confirm that ER-low positive BCs present aggressive biology and clinical characteristics similar to that usually described for ER-negative BC, in line with what was previously reported in literature^6,7,12^.

All patients included in our study were treated with neoadjuvant or adjuvant chemotherapy and no difference in the type of chemotherapy regimens applied in the two subgroups was reported. This highlights the fact that ER-low-positive tumors are usually treated in clinical practice similarly to ER-negative BCs.

Only a small number of patients in our study cohort received endocrine therapy, including a limited number of patients with ER-negative BCs (*N* = 13, 4%) who received endocrine treatment due to a previous or synchronous second ER-positive BC or ER-positive residual disease after neoadjuvant chemotherapy. In the ER-low positive subgroup, only 6 patients (14%) received adjuvant endocrine therapy. This can be partially explained by the fact that a significant number of patients were treated before the publication of ASCO/CAP 2010 guidelines that lowered the ER cut-off from 10 to 1%. Furthermore, in clinical practice, the use of adjuvant endocrine treatment of ER-low positive BC patients still remains debated. In fact, the 2020 ASCO/CUP guidelines update points out that even if ER-low positive BC patients should be considered eligible for endocrine treatment, there is only limited data suggesting the overall benefit of endocrine therapies in this subgroup. In light of growing evidence of the impact of endocrine treatment on patient quality of life and its toxicities^13^, potential benefits and risks in this subgroup of patients are usually carefully weighed and a significant number of ER-low positive BC patients do not receive adjuvant endocrine treatment in clinical practice. Nevertheless, a potential negative prognostic impact for ER-low positive BC patients not receiving adjuvant endocrine treatment cannot be completely ruled out based on available data and might potentially have affected study results. In this study, patients with ER-negative and ER-low positive BCs showed similar long-term outcomes, both in terms of iRFS and DRFS, and no significant difference was observed. Most studies comparing the long-term prognosis of ER-negative and ER-low positive BCs reported similar results^6–8^, while only two studies described a numerically better disease-free survival in the ER-low positive population as compared to the ER-negative population, but did not test statistically this result^14,15^. Similarly, 5-years OS was not significantly different in the two cohorts of patients (82.3% in the ER < 1% and 76.7% in the ER1–9%, *p* = 0.800), consistently with what was reported by most other studies (Table 3)^4,7,16,17^. The low number of patients in our study who received adjuvant hormonal therapy did not allow to draw conclusions about the impact of endocrine treatment in ER-low positive BC. This hypothesis must be tested using properly controlled data sets.

**Table 3:** Studies evaluating survival outcomes of ER-low positive BCs as compared to ER-negative BCs (only studies reporting ER expression in terms of % of ER-positive tumor cells and directly comparing the survival outcomes of ER low positive BC patients with either ER-negative or ER-positive subgroups are here reported; studies reporting ER expression as Allred score or *H*-score were not included).

	ER subgroups	Patients in each subgroup	Relapse outcome definition	Relapse outcome %	Relapse outcome hazard ratio (95% CI)	Relapse outcome *p*-value	Timepoint of OS evaluation	% OS at timepoint	OS hazard ratio (95% CI)	OS *p*-value
Iwamoto et al. (2012) ^4^	ER < 1%	183^a^	NR	NR	NR	NR	8 year- OS	75%	NR	Ref
	ER1–9%	25	NR	NR	NR	NR	8 year- OS	80%	NR	*p* = 0.21
Raghav et al. (2013) ^16^	ER < 1%	897	3 year-RFS	64%	Ref	Ref	3 year-OS	79%	Ref	Ref
	ER1–5%	241	3 year-RFS	67%	1.11 (0.88–1.41)	*p* = 0.37	3 year-OS	81%	1.10 (0.84–1.44)	*p* = 0.47
	ER6–10%	119	3 year-RFS	77%	0.92 (0.65–1.30)	*p* = 0.64	3 year-OS	88%	0.88 (0.6–1.31)	*p* = 0.54
Balduzzi et al. (2014) ^17^	ER < 1%	1300	5 year-DFS	74%	1.40 (0.92–2.12)	*p* = 0.115	5 year-OS	86%	1.50 (0.82–2.77)	*p* = 0.192
	ER1–10%	124	5 year-DFS	79%	Ref ^b^	Ref ^b^	5 year-OS	90%	Ref ^b^	Ref ^b^
Yi et al. (2014) ^24^	ER < 1%	1625^a^	RFS (no timepoint)	NR	1.2 (0.9–1.7)	*p* = 0.2	OS (no timepoint)	NR	1.5 (1.1–2)	*p* = 0.02
	ER1–9%	250	RFS (no timepoint)	NR	Ref ^b^	Ref ^b^	OS (no timepoint)	NR	Ref ^b^	Ref ^b^
Fujii et al. (2017) ^7^	ER < 1%	932	TTR (no timepoint)	NR	1.64 (1.34–1.99)^c^	*p* < 0.001^c^	OS (no timepoint)	NR	2.07 (1.67–2.58)^c^	*p* < 0.001^c^
	ER1–9%	171	TTR (no timepoint)	NR	1.92 (1.40–2.62)^c^	*p* < 0.001^c^	OS (no timepoint)	NR	2.35 (1.66–3.32)^c^	*p* < 0.001^c^

DFS disease-free survival, *ER* estrogen receptor, *HR* hazard ratio, OS overall survival, *RFS* recurrence-free survival, *TTR* time to recurrence.

^a^HER2-positive BC included.

^b^Hazard ratio and *p*-value of multivariate analysis.

^c^Hazard ratio and *p*-value of univariate analysis; ER ≥ 10% considered as reference.

In the treatment of early BC, the use of neoadjuvant therapy offers the opportunity to assess chemotherapy sensitivity and luminal BCs are well-known to present lower pCR rates as compared to triple-negative BCs^18^. In our study cohort, ER-low positive BC patients receiving neoadjuvant treatment showed a pCR rate of 44% (despite high rates of locally advanced BC), in line with that observed in the ER-negative cohort. Moreover, this result is in line with historical pCR rates reported for triple-negative BCs treated with neoadjuvant chemotherapy; in fact, a 33.6% pCR rate has been reported by Cortazar et al. for triple-negative BC in a large metanalysis^18^ and a recent metanalysis reported a 46.9% pCR rate in triple-negative BC patients treated with an anthracycline- and taxane-based neoadjuvant chemotherapy with or without the addition of carboplatin^19^. These findings are also consistent with what was previously reported in the few other studies that have described the response to neoadjuvant chemotherapy in ER-low positive BCs, which have reported pCR rates ranged between 25 and 37% (Table 4)^6–8,20^.

**Table 4:** Studies evaluating pathological complete response (pCR) rates in ER-low positive BCs treated with neoadjuvant chemotherapy (studies reporting ER expression as Allred score or *H*-score were also included).

	ER subgroups	Number of Patients in each subgroup	% pCR	OR (95%CI)/*p*-value
Gloyeske et al. (2014)^20^	ER-low positive (*H*-scores of 1–100)	18	33%	–
Fujii et al. (2017)^7^	ER < 1%	932	26%	0.95 (0.64–1.40)/*p* = 0.792
	ER1–9%	171	28%	Ref
	ER ≥ 10%	3055	7%	–
Landmann et al. (2018)^8^	ER-negative (*H*-score = 0)	141	26%	*p* = 0.1722
	Low ER^+^ (*H*-score 1–100)	41	37%	Ref
	Moderate ER^+^ (*H*-score 101–200)	47	11%	–
	High ER^+^ (*H*-score 201–300)	98	4%	–
Ohara et al. (2019)^6^	ER < 1%	32	41%	*p* = 0.296
	ER1–9%	16	25%	Ref
	ER ≥ 10%	108	7%	–

Overall, these results highlight that ER-low positive BC present a substantially different response to neoadjuvant treatment as compared to classical ER-positive BCs, which usually present low pCR rates ranging between 7 and 16% according to grade^18,21^.

Moreover, the achievement of pCR showed a similar impact on long-term outcomes in the ER-low positive and ER-negative subgroups. Patients with ER-low positive BCs who achieved pCR with neoadjuvant chemotherapy (44%) had an optimal long-term outcome (5-year iRFS 100%) without receiving any adjuvant endocrine treatment.

This study has some limitations which should be acknowledged. First, it is a retrospective monocentric study and its results should therefore be considered as exploratory and hypothesis-generating. Second, the number of ER-low positive patients is limited, consistently with the well-known rarity of this entity, not allowing subset analyses based on adjuvant endocrine therapy. Third, ER status reported in patient charts was used for classification in subgroups. Nevertheless, it also presents some major strengths. This is one of the very few studies reporting data for ER-low positive BC patients treated with neoadjuvant chemotherapy and the size of this subgroup is relatively large as compared to other published series. Moreover, even if ER-low positive cases were not systematically reviewed to confirm ER expression levels the large majority of these cases have been primarily evaluated by breast-dedicated pathologists from a single pathology unit, therefore assuring consistency in ER evaluation. In addition, all ER-low positive cases with available slides (88%) were reviewed to confirm internal control staining.

Early ER-low positive breast cancers are a rare subtype of tumor that shows similar clinical behavior and similar response to neoadjuvant chemotherapy to triple-negative breast cancer. Taken together, these findings add to the existing evidence suggesting the use of a 10% cut-off to define ER-positivity in breast cancer.

## Methods

### Patient cohort

All consecutive patients diagnosed with stage I–III BC with ER expression <10% by IHC and HER2-negative according to current ASCO/CAP recommendations treated with neoadjuvant and/or adjuvant chemotherapy at Istituto Oncologico Veneto of Padova between January 2000 and April 2019 were included. Demographic, clinicopathologic, and treatment data were retrospectively collected from medical charts. ER expression on pretreatment core biopsy specimen was collected for patients treated with neoadjuvant chemotherapy, while ER expression on the surgical specimen was collected for patients receiving adjuvant chemotherapy.

### Pathologic evaluation

The IHC protocol used for ER analysis by the Pathology Department of Azienda Ospedaliera di Padova, the reference pathology department for the Istituto Oncologico Veneto for BC patients, before and after January 2012 is here detailed. Between 2000 and 2012 IHC staining of formalin-fixed paraffin-embedded (FFPE) tissue was performed using SP1 (790-4325, Ventana Medical System, Tucson AZ, pre-diluted) for ER, 1E2 (790-4296, Ventana Medical System, Tucson AZ, pre-diluted) for PR and 4B5 (790-2991, Ventana Medical System, Tucson AZ, pre-diluted) for HER2. For ER, heat-induced antigen retrieval was done using Cell Conditioning 1 for 36 min and slides were then incubated at 37 °C for 16 min.

Since 2012 included, IHC staining was performed using 6F11 (PA0153, Leica Biosystems Newcastle, Newcastle UK, pre-diluted) for ER, 16 (PA0322, Leica Biosystems Newcastle, Newcastle UK, 1:100) for PR and CB11 (NCL-CB11, Leica Biosystems Newcastle, Newcastle UK, pre-diluted) per HER2. For ER, slides were pretreated with EDTA for 20 min and then incubated for 15 min at room temperature.

All ER-low positive cases with available slides (37 cases out of 42 ER-low cases) were reviewed by a dedicated breast cancer pathologist (MLM). All internal controls were double-checked for staining which was confirmed in all cases and concordance for ER-low expression was 95%.

Patients were categorized into two groups according to IHC ER expression reported in medical records, which was used for clinical decisions: ER < 1% or ER1–9%.

### Outcome definition

pCR was defined as a complete absence of infiltrating cancer cells in the breast and locoregional lymph nodes after neoadjuvant chemotherapy (ypT0/is, ypN0). iRFS was defined as the time from diagnosis to invasive relapse (locoregional or distant), death, or last follow-up. DRFS was defined as the time from diagnosis to distant relapse, death, or last follow-up. OS was defined as the time from diagnosis to death or last follow-up.

### Statistical analysis

Statistical analysis was performed using R 3.6.1^22^. The clinicopathological characteristics of the two cohorts were summarized using standard descriptive statistics. Association between patients’ groups, clinicopathological characteristics, and pCR were evaluated using chi-square test, Fisher’s exact test, Mann–Whitney U test, or Student *t*-test according to the type of variable considered. Kaplan–Meier method was used to estimate iRFS, DRFS, and OS and their 95% CI. For the evaluation of prognostic factors, hazard ratio (HR) and 95% CI were calculated with the Cox proportional hazard regression model (univariate and multivariate). All *p*-values were two-sided, with significance levels set at *p* < 0.05.

The study was approved by the ethics committee of Istituto Oncologico Veneto and all relevant ethical regulations have complied. Informed consent was obtained from all participants.

### Reporting summary

Further information on research design is available in the Nature Research Reporting Summary linked to this article.

## Supplementary information

Supplementary Information

Reporting Summary

## Data Availability

The data generated and analyzed during this study are described in the following data record: 10.6084/m9.figshare.14806575^23^. The data underlying the claims of this article (demographic, clinicopathologic, and treatment data retrospectively collected from medical charts; ER expression on pretreatment core biopsy specimens for patients treated with neoadjuvant chemotherapy; ER expression on surgical specimens for patients receiving adjuvant chemotherapy) are contained in the file ‘TNBC_ERlow_Padova.txt’. This file is not publicly available for the following reason: data contain information that could compromise research participant privacy and informed consent to share participant-level data was not obtained prior to or during data collection. However, the data can be made available upon request to the corresponding author.

## Code availability

The R software (version 3.6.1) codes used during the current study are available from the corresponding author on reasonable request.

## References

[1] Gradishar WJ (2020). Breast cancer, version 3.2020, NCCN clinical practice guidelines in oncology. J. Natl Compr. Canc. Netw..

[2] Hammond MEH (2010). American society of clinical oncology/college of American pathologists guideline recommendations for immunohistochemical testing of estrogen and progesterone receptors in breast cancer. J. Clin. Oncol..

[3] Harvey JM, Clark GM, Osborne CK, Allred DC (1999). Estrogen receptor status by immunohistochemistry is superior to the ligand-binding assay for predicting response to adjuvant endocrine therapy in breast cancer. J. Clin. Oncol..

[4] Iwamoto T (2012). Estrogen receptor (ER) mRNA and ER-related gene expression in breast cancers that are 1% to 10% ER-positive by immunohistochemistry. J. Clin. Oncol..

[5] Cheang MCU (2015). Defining breast cancer intrinsic subtypes by quantitative receptor expression. Oncologist.

[6] Ohara AM (2019). PAM50 for prediction of response to neoadjuvant chemotherapy for ER-positive breast cancer. Breast Cancer Res. Treat..

[7] Fujii T (2017). Revisiting the definition of estrogen receptor positivity in HER2-negative primary breast cancer. Ann. Oncol..

[8] Landmann A (2018). Low estrogen receptor (ER)-positive breast cancer and neoadjuvant systemic chemotherapy. Am. J. Clin. Pathol..

[9] Allison KH (2020). Estrogen and progesterone receptor testing in breast cancer: ASCO/CAP guideline update. J. Clin. Oncol..

[10] Conte PF (2020). Phase III randomized study of adjuvant treatment with the ANTI-PD-L1 antibody avelumab for high-risk triple negative breast cancer patients: The A-BRAVE trial. J. Clin. Oncol..

[11] Voorwerk L (2019). Immune induction strategies in metastatic triple-negative breast cancer to enhance the sensitivity to PD-1 blockade: the TONIC trial. Nat. Med..

[12] Deyarmin B (2013). Effect of ASCO/CAP guidelines for determining ER status on molecular subtype. Ann. Surg. Oncol..

[13] Ferreira AR (2019). Differential impact of endocrine therapy and chemotherapy on quality of life of breast cancer survivors: a prospective patient-reported outcomes analysis. Ann. Oncol..

[14] Prabhu JS (2014). A majority of low (1-10 %) ER positive breast cancers behave like hormone receptor negative tumors. J. Cancer.

[15] Viale G, Regan MM, Maiorano E, Mastropasqua MG, Orto PD (2020). Prognostic and predictive value of centrally reviewed expression of estrogen and progesterone receptors in a randomized trial comparing letrozole and tamoxifen adjuvant therapy for postmenopausal early breast cancer: BIG 1. J. Clin. Oncol..

[16] Raghav KPS (2012). Impact of low estrogen/progesterone receptor expression on survival outcomes in breast cancers previously classified as triple negative breast cancers. Cancer.

[17] Balduzzi A (2014). Survival outcomes in breast cancer patients with low estrogen/progesterone receptor expression. Clin. Breast Cancer.

[18] Cortazar, P. et al. Pathological complete response and long-term clinical benefit in breast cancer: The CTNeoBC pooled analysis. *Lancet*10.1016/S0140-6736(13)62422-8 (2014). 10.1016/S0140-6736(13)62422-8 24529560

[19] Poggio F (2018). Platinum-based neoadjuvant chemotherapy in triple-negative breast cancer: a systematic review and meta-analysis. Ann. Oncol..

[20] Gloyeske, N. C., Dabbs, D. J. & Bhargava, R. Low ER + breast cancer is this a distinct group? *Am. J. Clin. Pathol.* 697–701, 10.1309/AJCP34CYSATWFDPQ (2014). 10.1309/AJCP34CYSATWFDPQ 24713741

[21] Guarneri V (2006). Prognostic value of pathologic complete response after primary chemotherapy in relation to hormone receptor status and other factors. J. Clin. Oncol..

[22] R Core Team. *R: A Language and Environment for Statistical Computing*. https://www.R-project.org/ (R Foundation for Statistical Computing, Vienna, Austria, 2017).

[23] Dieci, M. V. et al. Metadata record for the article: impact of estrogen receptor levels on outcome in non-metastatic triple negative breast cancer patients treated with neoadjuvant/adjuvant chemotherapy. figshare 10.6084/m9.figshare.148065. (2021). 10.1038/s41523-021-00308-7 PMC8329161 34341356

[24] Yi M (2014). Which threshold for ER positivity? a retrospective study based on 9639 patients. Ann. Oncol..

