# Supplementary figures and images for: Impact of estrogen receptor levels on outcome in non-metastatic triple negative breast cancer patients treated with neoadjuvant/adjuvant chemotherapy
Source: NPJ Breast Cancer. 2021 Aug 2;7:101. doi: 10.1038/s41523-021-00308-7 (PMC8329161; doi:10.1038/s41523-021-00308-7)

**Supplementary Figure 1. Study cohort**

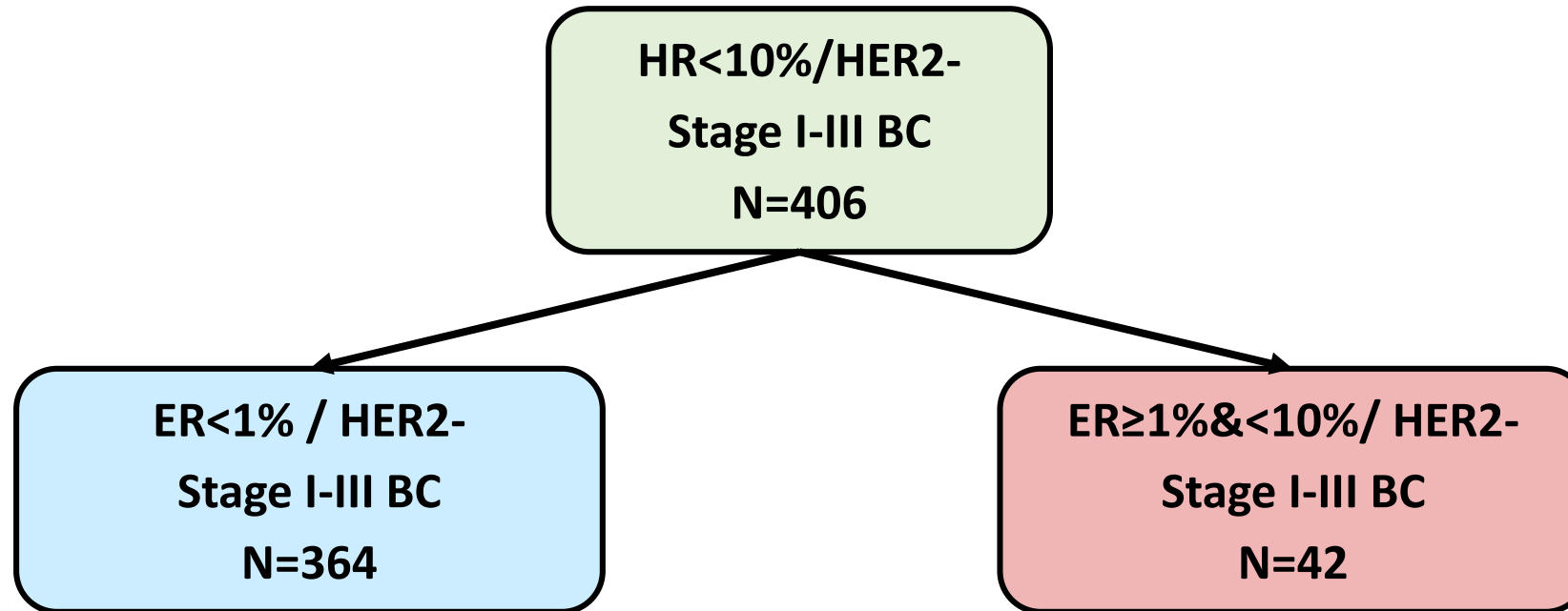

Supplement: Supplementary file 1 — Supplementary Information [file 41523_2021_308_MOESM1_ESM.pdf]
